# Glycerol Monolaurate Antibacterial Activity in Broth and Biofilm Cultures

**DOI:** 10.1371/journal.pone.0040350

**Published:** 2012-07-11

**Authors:** Patrick M. Schlievert, Marnie L. Peterson

**Affiliations:** 1 Department of Microbiology, University of Iowa, Iowa City, Iowa, United States of America; 2 Experimental and Clinical Pharmacology, University of Minnesota, Minneapolis, Minnesota, United States of America; The Scripps Research Institute, United States of America

## Abstract

**Background:**

Glycerol monolaurate (GML) is an antimicrobial agent that has potent activity against gram-positive bacteria. This study examines GML antibacterial activity in comparison to lauric acid, in broth cultures compared to biofilm cultures, and against a wide range of gram-positive, gram-negative, and non-gram staining bacteria.

**Methodology/Principal Findings:**

GML is ≥200 times more effective than lauric acid in bactericidal activity, defined as a ≥3 log reduction in colony-forming units (CFU)/ml, against *Staphylococcus aureus* and *Streptococcus pyogenes* in broth cultures. Both molecules inhibit superantigen production by these organisms at concentrations that are not bactericidal. GML prevents biofilm formation by *Staphylococcus aureus* and *Haemophilus influenzae*, as representative gram-positive and gram-negative organisms, tested in 96 well microtiter plates, and simultaneously is bactericidal for both organisms in mature biofilms. GML is bactericidal for a wide range of potential bacterial pathogens, except for *Pseudomonas aeruginosa* and *Enterobacteriaceae*. In the presence of acidic pH and the cation chelator ethylene diamine tetraacetic acid, GML has greatly enhanced bactericidal activity for *Pseudomonas aeruginosa* and *Enterobacteriaceae*. Solubilization of GML in a nonaqueous delivery vehicle (related to K-Y Warming®) enhances its bactericidal activity against *S. aureus*. Both R and S, and 1 and 2 position lauric acid derivatives of GML exhibit bactericidal activity. Despite year-long passage of *Staphylococcus aureus* on sub-growth inhibitory concentrations of GML (0.5 x minimum bactericidal concentration), resistance to GML did not develop.

**Conclusions/Significance:**

GML may be useful as a broad-spectrum human or animal topical microbicide and may be useful as an environmental surface microbicide for management of bacterial infections and contamination.

## Introduction

Most bacterial pathogens initiate human illnesses from intact or damaged mucosal or skin surfaces. Many of these pathogens are acquired from other persons or animals, from endogenous sources, or from a myriad of environmental sources. Once in humans, pathogens colonize surfaces primarily as biofilms of organisms, defined as thin-films of organisms attached to host tissues, medical devices, and other bacteria through complex networks of polysaccharides, proteins, and nucleic acids [1]. These bacteria may also exist as planktonic (broth) cultures in some host tissue environments, such as the bloodstream and mucosal secretions. Similarly, these potential pathogens may exist as either biofilms or planktonic cultures in a myriad of non-living environments [1]. A major objective of the present studies is to assess the ability of a fatty acid monoester, glycerol monolaurate (GML), to inhibit growth and exotoxin production by bacteria as grown in biofilms and planktonic cultures.

GML is a generally recognized as safe natural compound by the Food and Drug Administration (FDA) that exhibits potent antibacterial activity against gram-positive cocci, and *Bacillus anthracis* [2], [3]. Unlike most antibiotics which have single bacterial targets for antibacterial activities, GML appears to target many bacterial surface signal transduction systems nonspecifically through interaction with plasma membranes [2], [3], [4]. GML also inhibits exotoxin production by gram-positive bacteria at GML concentrations that do not inhibit bacterial growth [2], [3], [4]. These properties are shared with the antibiotic clindamycin, a protein synthesis inhibitor [5]. GML is also virucidal for enveloped viruses, apparently through its ability to interfere with virus fusion with mammalian cells, and through GML's ability to prevent mucosal inflammation required for some viruses to penetrate mucosal surfaces [6], [7], [8].

In 2005, Ruzin and Novick suggested that the ability of GML to inhibit exotoxin production by *Staphylococcus aureus* resulted from its cleavage to lauric acid [9]. These authors demonstrated that lauric acid was identical in activity to GML. These data are surprising for at least two reasons: 1) GML can be degraded by esterases of *Staphylococcus aureus*, including the major esterase glycerol ester hydrolase, to glycerol and lauric acid. When degraded to lauric acid, GML loses antibacterial activity and its ability to inhibit exotoxin production [2]; and 2) many bacteria, such as streptococci, do not make esterases and thus cannot cleave GML to lauric acid. Yet, GML is even more highly antibacterial to these organisms, with highly significant ability to inhibit exotoxin production at non-growth-inhibitory concentrations [2]. A second major objective of our study is to evaluate GML and lauric acid in side-by-side comparisons for bactericidal activity and their ability to prevent exotoxin production at non-growth-inhibitory concentrations.

Multiple forms of GML exist or can be synthesized. Previously, we tested dodecyl glycerol (DDG, glycerol ether-linked to lauric acid) compared to GML for antibacterial activity and ability to inhibit exotoxin production independently of bactericidal activity [10]. Both DDG and GML inhibit *Staphylococcus aureus* growth, with both being either bacteriostatic or bactericidal dependent concentration. However, GML inhibits exotoxin production at non-growth-inhibitory concentrations, whereas DDG does not inhibit exotoxin production independent of growth inhibition [10]. Both R and S forms of GML and GML with lauric acid in the 1/3 and 2 positions exist. These molecules have not been tested for antimicrobial activity but are tested in this study.

We previously showed that *Escherichia coli* and *Salmonella minnesota* with their native lipopolysaccharide (LPS) layer cannot be inhibited from growing due to GML [2]. In contrast, a *Salmonella Minnesota* Re mutant, lacking the O side chain and much of the common core polysaccharide components of LPS, are easily killed by GML. This led us to propose that other gram-negative bacteria that have lipooligosaccharide (LOS) instead of *Enterobacteriaceae* intact LPS may be susceptible to GML. In vitro studies by us and others have shown that both *Gardnerella vaginalis* and *Helicobacter pylori*, with LOS instead of intact LPS, are highly susceptible to the bactericidal activity of GML [11]. These studies have been confirmed with in vivo studies in humans with demonstration of *Gardnerella vaginalis* susceptibility to GML [11].

The above studies with *Salmonella minnesota* suggest that agents that disrupt the LPS layer may increase the activity of GML. Such agents may include protonation of the bacterial surface by reduced pH to repel calcium and magnesium in the LPS layer, divalent cation chelators such as ethylene diamine tetraacetic acid (EDTA), and biocompatible non-aqueous GML delivery vehicles (such as K-Y Warming® gel) that intrinsically would interfere with the bacterial inner and outer membranes and LPS. Studies were undertaken to assess the efficacy of low pH and EDTA in amplification of GML activity against highly resistant *Enterobacteriaceae* and *Pseudomonas aeruginosa*, and a non-aqueous delivery vehicle for amplification of activity against *S. aureus.*

Our data from the present studies demonstrate that GML is bactericidal for aerobic and anaerobic gram-positive bacteria in broth and biofilm cultures, GML exhibits greater bactericidal activity than lauric acid, and all forms of GML exhibit antibacterial activity. Additionally, GML is bactericidal for gram-negative bacteria with LOS instead of LPS, but GML becomes bactericidal for naturally GML-resistant *Enterobacteriaceae* by addition of agents that disrupt the LPS layer. Gram-negative anaerobes are susceptible to GML. *Pseudomonas aeruginosa* appear to be the most resistant bacteria tested, but these organisms are killed by GML at pH 5.0–6.0.

## Results

### Comparison of GML and Lauric Acid Antimicrobial Activity

Previously, we demonstrated that β-hemolytic streptococci, lacking glycerol ester hydrolase (GEH), exhibit greater susceptibility to GML than *Staphylococcus aureus* which produce GEH [2]. This suggests that GML has greater antibacterial activity than lauric acid which with glycerol are the two major cleavage products of GML. However, a publication by Ruzin and Novick in 2000 suggests that the ability of GML to inhibit exotoxin production by *Staphylococcus aureus* is equivalent to inhibition of exotoxin production by lauric acid [9]. We thus compared GML and lauric acid for their antibacterial activity and ability to inhibit exotoxin production at concentrations that do not inhibit bacterial growth.

**Figure 1:**
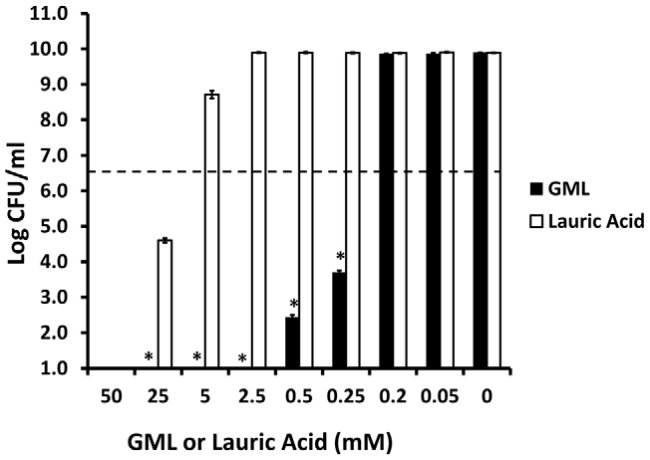
GML and lauric acid inhibition of *Staphylococcus aureus* MN8 growth. GML and lauric acid were incubated with approximately 5×10^6^ CFUs/ml of *Staphylococcus aureus* for 24 hours with shaking (200 revolutions/min) at 37°C in triplicate. Plate counts were used to determine CFUs/ml. Bars show standard deviations. Bactericidal activity was defined as the minimum concentration of agent required to reduce CFUs by ≥3 logs. * indicates GML mean significantly different from lauric acid mean with p<0.001. Dashed line indicates starting inoculum size.

**Figure 2:**
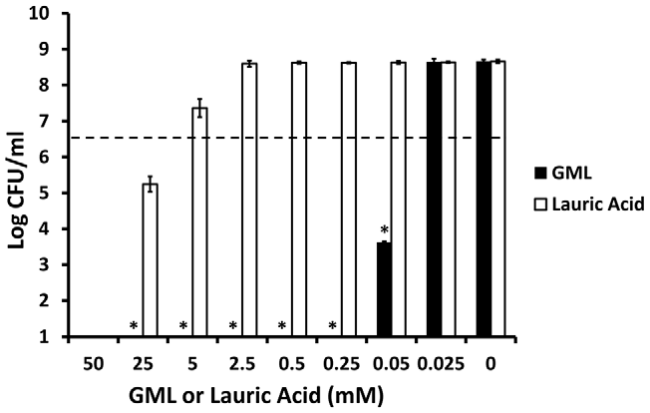
GML and lauric acid inhibition of *Streptococcus pyogenes* growth. GML and lauric acid were incubated with approximately 5×10^6^ CFU/ml of *Streptococcus pyogenes* for 24 hours stationary in the presence of 7% CO_2_ at 37°C in triplicate. Plate counts were used to determine CFUs/ml. Bars show standard deviations. Bactericidal activity was defined as the minimum concentration of agent required to reduce CFUs by ≥3 logs. * indicates GML mean significantly different from lauric acid mean with p<0.001. Dashed line indicates starting inoculum size.

GML was bactericidal at 200-fold lower concentrations than lauric acid when tested against *Staphylococcus aureus* MN8, a typical menstrual toxic shock syndrome (TSS) strain. GML was bactericidal, defined in this study as a ≥3 log reduction in CFUs/ml relative to starting inocula, at 0.25 mM, whereas lauric acid was bactericidal at 50 mM (Figure 1). When GML and lauric acid were tested against a scarlet fever strain of *Streptococcus pyogenes* (strain T25_3_curedT12), an organism that lacks GEH, GML exhibited 5-fold (0.05 mM) greater bactericidal activity (Figure 2) than when tested against *Staphylococcus aureus* MN8 (Figure 1). In contrast, there was no difference in bactericidal activity of lauric acid for either *Streptococcus pyogenes* or *Staphylococcus aureus* MN8 (Figures 1 and 2).

**Figure 3:**
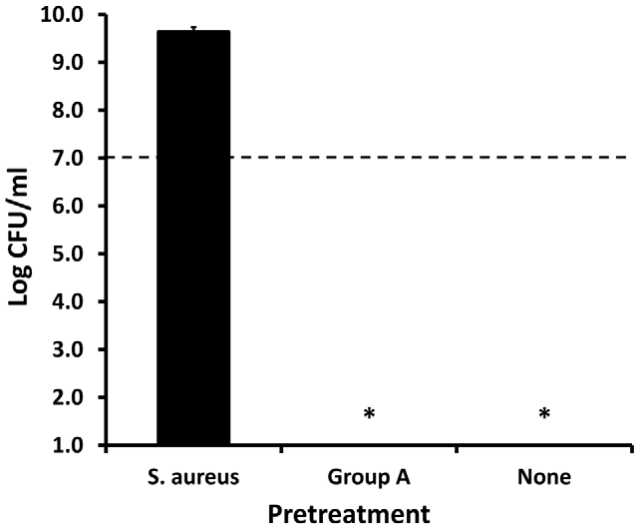
Ability of pretreatment of GML with *Staphylococcus aureus* MN8 spent culture fluids to reduce antimicrobial activity. *Staphylococcus aureus* MN8 or *Streptococcus pyogenes* T25_3_curedT12 sterile stationary phase culture fluids were incubated with GML (1000 μg) for 24 hours. Subsequently, treated GML was evaluated for antibacterial activity against *Staphylococcus aureus* MN8 in a 24 hour assay, in comparison to untreated GML. * indicates mean significantly reduced from starting inoculum mean at p<0.001. Dashed line indicates starting inoculum size.

Pretreatment of GML (1000 μg/0.4 ml Todd Hewitt broth) with 0.1 of stationary phase sterile culture fluid from *Staphylococcus aureus* MN8 overnight at 37°C eliminated its antibacterial activity against *Staphylococcus aureus* MN8 (Figure 3), whereas comparable treatment with *Streptococcus pyogenes* did not reduce GML antimicrobial activity.

**Figure 4:**
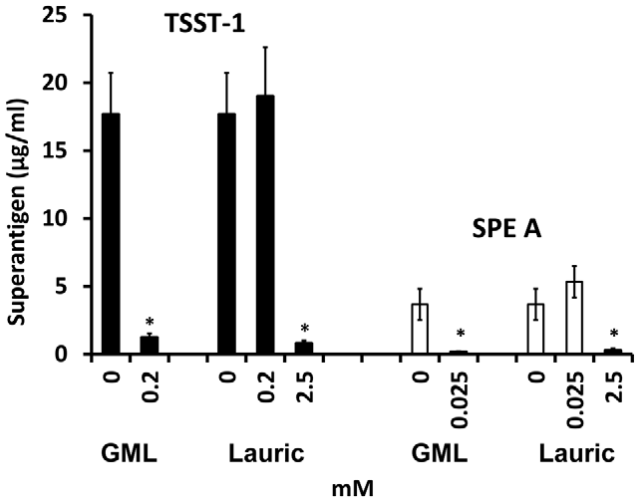
Comparison of GML and lauric acid for capacity to inhibit superantigen production. *Staphylococcus aureus* MN8 and *Streptococcus pyogenes* were cultured for 8 hours in the presence of GML or lauric acid. Toxic shock syndrome toxin-1 (TSST-1) production by *Staphylococcus aureus* MN8 and streptococcal pyrogenic exotoxin A (SPE A) production by *Streptococcus pyogenes* were quantified by Western immunoblot analysis. * indicates mean significantly reduced from the no GML mean at p<0.01.

Both GML and lauric acid significantly inhibited superantigen production by *Staphylococcus aureus* MN8 and *Streptococcus pyogenes* at sub-growth inhibitory concentrations (Figure 4). However, the GML concentration that was required for inhibition of exotoxin production in absence of growth inhibition was lower for both organisms than lauric acid.

Collectively, these data indicate that both GML and lauric acid have antibacterial activity and ability to inhibit exotoxin production at concentrations that do not inhibit bacterial growth. However, GML exhibits greater antibacterial activity than lauric acid.

### GML Bactericidal Activity in Biofilms

**Figure 5:**
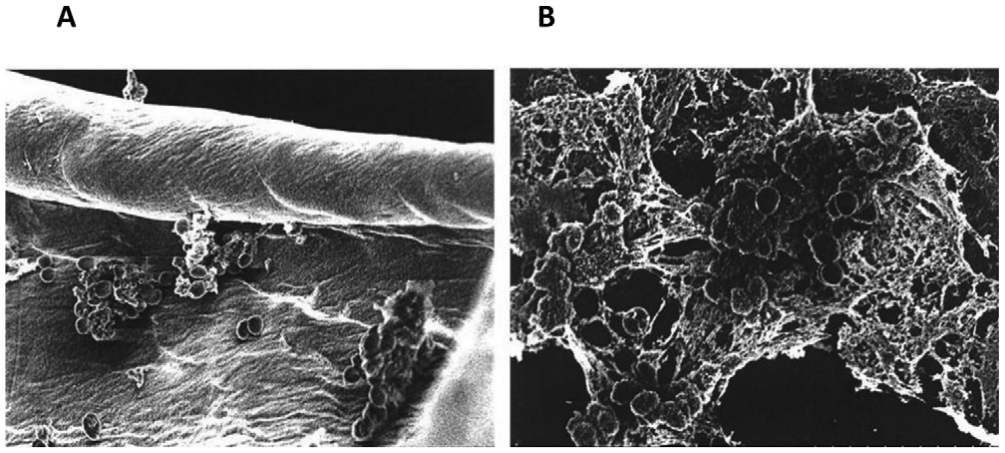
Community-associated *Staphylococcus aureus* 128 growing as biofilms on tampon fibers and cellulose acetate dialysis tubing. *Staphylococcus aureus* 128, a TSST-1^+^ organism at 10^7^/0.1 ml, was inoculated onto the inside of pre-wetted dialysis tubing that had been tied off on one end. A tampon was then inserted into the dialysis tubing and the tubing immersed under Todd Hewitt broth containing 0.8% agar. The open end of the dialysis tubing remained above the agar surface such that the only source of nutrients for the growing microbes was media absorbed across the dialysis tubing. Magnification of A was 6000x and B was 9000x. Both frames show organisms embedded in a complex matrix of extracellular material.

Many bacteria that are found on surfaces grow as biofilms [1], forming complex extracellular matrices (example in Figure 5). In the assay shown, a regular absorbency tampon was placed within dialysis tubing inoculated with community-associated (CA) methicillin-resistant *Staphylococcus aureus* (MRSA) 128 (TSST-1^+^), creating a tampon sac. [12] This tampon sac was submerged beneath Todd Hewitt broth containing 0.8% agar for 16 hours and then evaluated for biofilm formation on both tampon fibers (Figure 5A) and dialysis tubing (Figure 5B). *Staphylococcus aureus* 128 grew as complex biofilms on both the tampon fibers and on the dialysis tubing. It is our experience that cellulose acetate provides one of the best substrates for biofilm formation.

**Figure 6:**
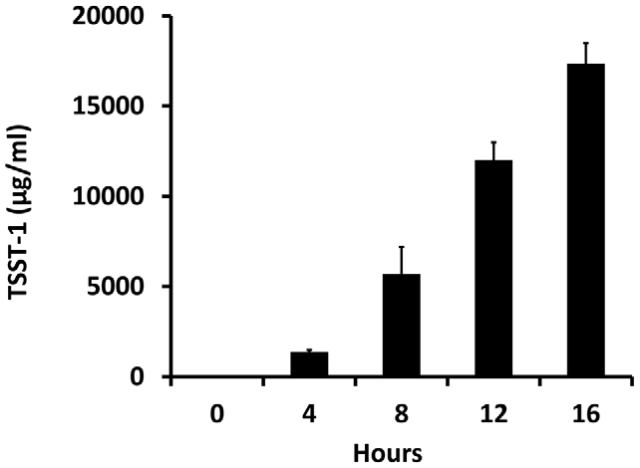
TSST-1 production in biofilms in tampon sacs. *Staphylococcus aureus* MN8 (10^7^/0.1 ml) were placed inside dialysis tubes that had been tied off on one end, and then tampons were inserted. The tampon sacs were immersed beneath Todd Hewitt 0.8% agar such that the open end of the dialysis tubing remained above the surface. The agar was allowed to solidify, and the tampons sacs were incubated for indicated times. TSST-1 was measured within the dialysis tube by knowing the pre- and post-weight of the tampons, eluting TSST-1 with distilled water, and quantifying TSST-1 by Western immunoblot analysis.

A previous study suggested that pulsed-field gel electrophoresis type USA200 strains of *Staphylococcus aureus* are attenuated in virulence in part due to a mutation in the accessory gene regulator (*agr*) global regulatory system [13]. These strains are the common causes of menstrual TSS and include strains MN8 and 128. In previous tests of over 5000 such USA200 strains, all produced 3.5 to 20 μg/ml of TSST-1 in broth cultures (unpublished data). It is estimated that 0.1 μg of TSST-1 is sufficient to cause TSS, indicating that these strains produce 350–2000 TSS-inducing doses/ml culture [14]. We evaluated the ability of *Staphylococcus aureus* 128 to produce TSST-1 in biofilms as grown in tampon sacs (Figure 6). In these cultures by 4 hours post infection, TSST-1 amounts exceeded 1300 μg/ml (13,000 TSS inducing doses/ml) and by 16 hours were at 18,000 μg/ml (180,000 TSS-inducing doses/ml). By 16 hours the tampons had absorbed 5.5 ml of medium, indicating 99,000 μg total TSST-1 (990,000 TSS-inducing doses). Comparatively, *Staphylococcus aureus* 128 produces 5 μg/ml in overnight Todd Hewitt broth cultures. Collectively, these data indicate that despite a mutation in *agr*, TSST-1 is produced in exceptionally high amounts in biofilms. This study has been repeated with strains MN8 and CDC587 with comparable results. In tampon sacs that contained 5% GML (w/w), no TSST-1 was detected (data not shown).

**Figure 7:**
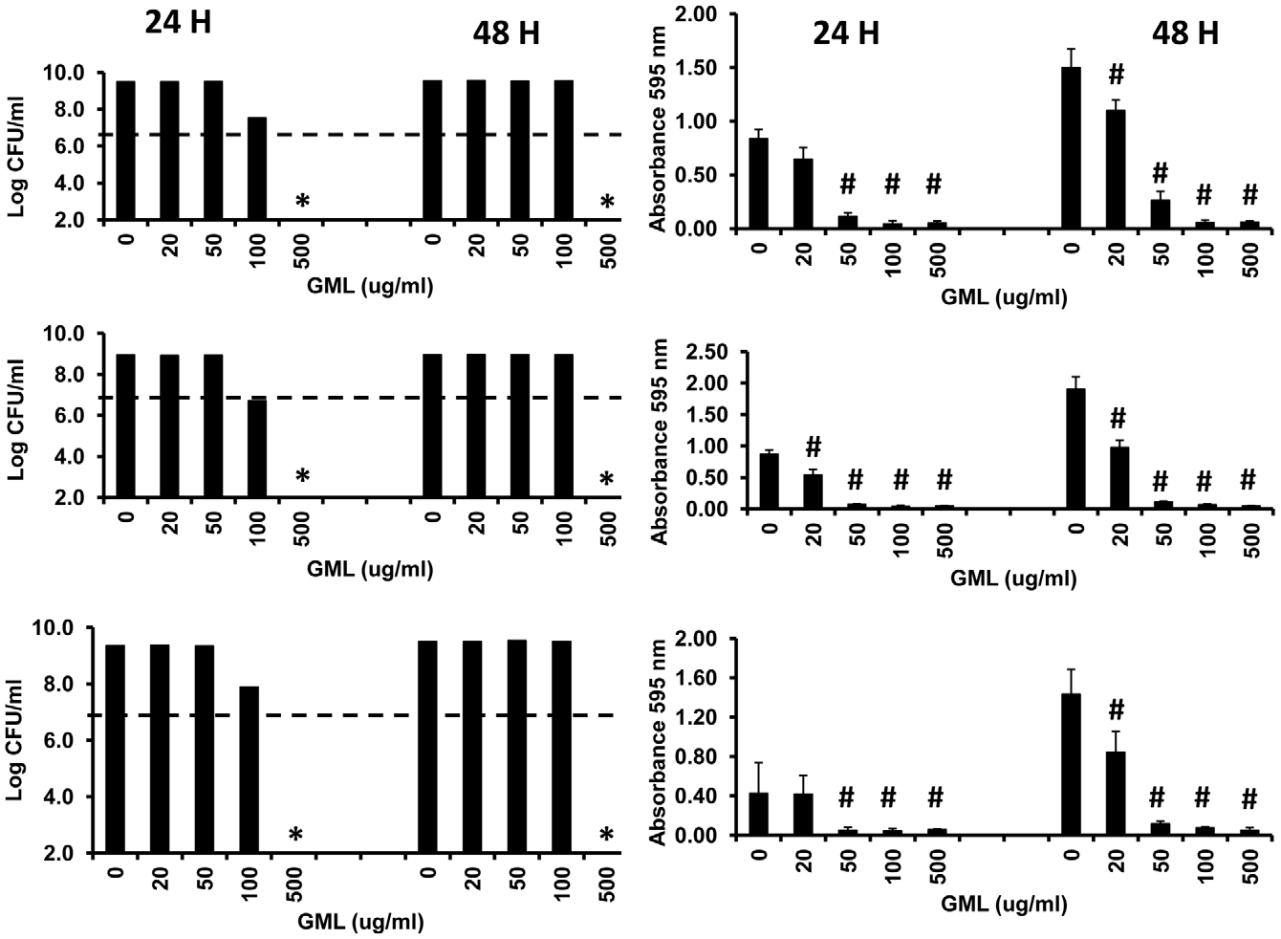
Effect of GML on three *Staphylococcus aureus* strains cultured in biofilms. *Staphylococcus aureus* strains methicillin-sensitive MN8, community-associated methicillin-resistant MNWH, and community-associated methicillin-resistant MW2 were cultured for 24 and 48 hours in 96 well microtiter plates. In one set of three wells for each microbe, the wells were agitated 3 times by pipetting up and down. CFU/ml were determined on supernates then removed from the wells. * indicates significant mean reduction compared to starting inoculum mean at p<0.001. Dashed line indicates starting inoculum size. Subsequent to removing unbound cells and then washing wells three times with phosphate-buffered saline, the wells were treated with crystal violet for 30 min. The wells were next washed three times with phosphate-buffered saline to remove unbound crystal violet. Finally, wells were treated with ethanol to solubilize biofilm-associated crystal violet. Absorbances at 595 nm were determined by an ELISA reader. # indicates significant mean reduction in absorbance at 595 nm wavelength between no GML and GML-treated wells at p<0.01.

**Figure 8:**
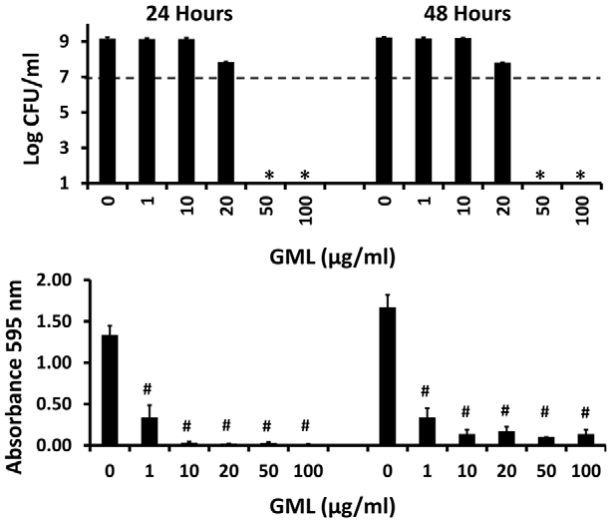
Effect of GML on non-typable *Haemophilus influenzae* cultured in biofilms. *Haemophilus influenzae* was cultured for 24 and 48 hours in a 96 well microtiter plate. In one set of three wells for the microbe, the wells were agitated 3 times by pipetting up and down and then supernates removed for plate counting to determine CFUs/ml. * indicates significant mean reduction compared to starting inoculum mean at p<0.001. Dashed line indicates starting inoculum size. Subsequent to removing bacterial cells and washing three times with phosphate-buffered saline, the wells were treated with crystal violet for 30 min. The wells were then washed three times with phosphate-buffered saline to remove unbound crystal violet. Finally, the wells were treated with ethanol to solubilize biofilm-associated crystal violet. Absorbances at 595 nm were determined by an ELISA reader. # indicates significant mean reduction in absorbance at 595 nm wavelength between no GML and GML-treated wells at p<0.01.

The most straightforward way to quantify biofilm formation is the use of 96 well microtiter plates incubated stationary at 37°C with test bacteria and then assaying microbial components retained on the wells after washing [15]. This assay was used to assess the ability of GML to interfere with biofilm formation by three strains of *Staphylococcus aureus* (Figure 7) and a non-typable *Haemophilus influenzae* (Figure 8). All three *Staphylococcus aureus* strains were completely growth-inhibited by GML only at 500 μg/ml at both 24 and 48 hours. In contrast, at 10 times lower GML concentrations than necessary to inhibit bacterial growth, biofilm formation was significantly inhibited as measured by reduced crystal violet straining of retained biofilms materials in wells of the microtiter plates. GML also prevented biofilm formation by non-typable *Haemophilus influenzae* (Figure 8). GML was bactericidal at GML concentrations of 50 μg/ml, but significantly prevented biofilm formation at concentrations of 1.0 μg/ml.

**Figure 9:**
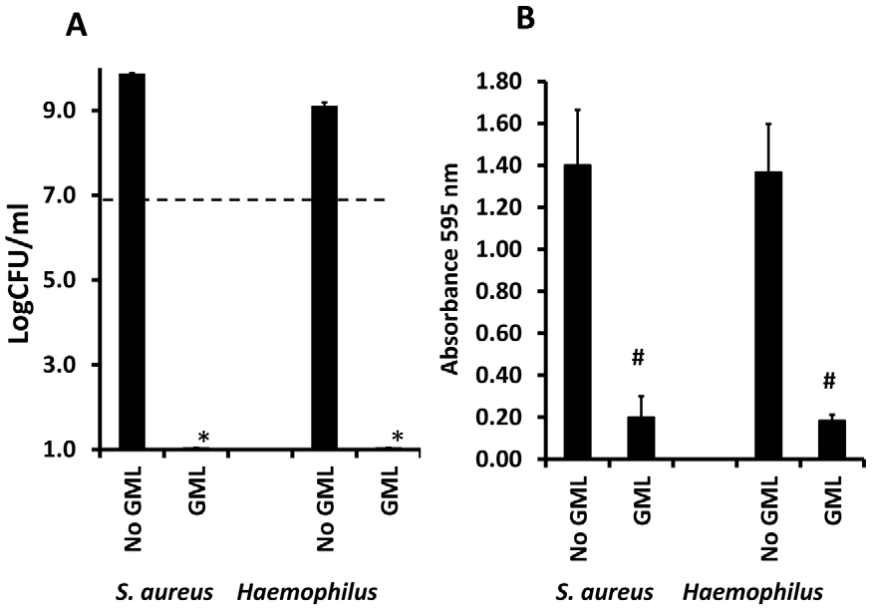
Ability of GML to remove pre-formed biofilms. *Staphylococcus aureus* MN8 and nontypable *Haemophilus influenzae* were grown for 48 hours in 96 well microtiter plates to allow biofilm formation. CFUs/ml were determined after suspension of organisms (A). Subsequently, the wells were treated or not treated with GML (500 μg/ml) for 60 min, and then CFUs/ml determined again (A). * indicates significant mean reduction compared to starting inoculum mean at p<0.001. Dashed line indicates starting inoculum size. Wells were stained with crystal violet after removal of non-adherent bacteria or after removal of non-adherent bacteria and treatment with GML, followed by removal of unbound crystal violet and determination of absorbance at 595 nm (B). # indicates significant mean reduction in absorbance at 595 nm wavelength between no GML and GML-treated wells at p<0.01.

Side-by-side wells that were not treated with GML and that had high absorbances at 595 nm at 48 hours received GML (500 μg/ml) for 60 min at 37°C. Subsequently, CFUs/ml were determined, after agitation with a pipette wells were then washed three times with PBS, and crystal violet straining determined to assess if GML could removed previously formed biofilms and simultaneously kill the microbes. After 48 hours, GML (500 μg/ml) was highly effective in both killing *Staphylococcus aureus* MN8 and non-typable *Haemophilus influenzae* by 60 min addition to the cultures (Figure 9A). Similarly, GML (500 μg/ml) nearly completely removed biofilm material attached to the wells after 48 hours as noted by loss of crystal violet straining (Figure 9B).

### Lack Resistance to GML by Staphylococcus aureus

Antibiotic resistance in bacteria may occur with treatment of patients with sub-optimal antibiotic concentrations or with prolonged antibiotic therapy. We tested *Staphylococcus aureus* MN8 for one year with sub-inhibitory (0.5 x minimum bactericidal concentration [MBC]) GML concentrations (50 μg/ml) to evaluate the potential to develop resistance. Our hypothesis was that GML has many plasma membrane targets, including interference with signal transduction, and with multiple GML bacterial targets, resistance to GML is unlikely. We chose to study *Staphylococcus aureus* for two reasons: 1) the organism has demonstrated rapid development of resistance to many antibiotics, and 2) the organism produces GEH [2], where up-regulation of GEH production could lead to resistance. Each week *Staphylococcus aureus* MN8 was transferred to GML Todd Hewitt agar plates containing 50 μg/ml GML (0.5 MBC) and cultured 48 hours; *Staphylococcus aureus* MN8 will grow on the plates in 48 hours. Colonies (50–200) that grew on the Todd Hewitt agar plates were scraped off and passed weekly onto new Todd Hewitt plates containing GML (50 μg/ml). Each week the organisms that grew on the GML (50 μg/ml) plates were also transferred onto GML (100 μg/ml) (1×MBC) plates at approximately 1×10^9^ CFU/plate, where the organism cannot grow in 48 hours. Over the entire year of testing, no GML-resistant *Staphylococcus aureus* MN8, that were able to grow on the Todd Hewitt plates containing GML (100 μg/ml), were obtained. Additionally, the plates that had 50 μg/ml GML weekly were also placed at 4°C to allow GML to crystallize. Through measurement of non-crystallizing zones around individual colonies, which is reflective of GEH cleavage of GML, no increased zones of GEH cleavage of GML were seen at any time point.

### Tests of Bactericidal Activities of R Versus S and 1 Versus 2 Position Lauric Acid Forms of GML

**Figure 10:**
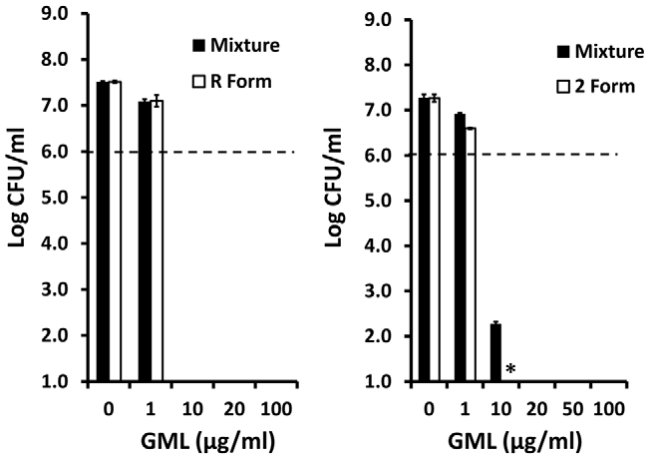
Ability of R versus S and 1/3 versus 2 forms of GML to inhibit growth of *Streptococcus pyogenes* 594. R form and mixture of R and S forms of GML and 2 position lauric acid versus mixture of 1/3 and 2 position lauric of GML were incubated with *Streptococcus pyogenes* 594, a highly susceptible organism to GML, for 24 hours. Plate counts were used to determine GML killing. * indicates the mean for the 2 form of GML is significantly different from the mixture at p<0.001. Dashed line indicates starting inoculum size.

Multiple forms of GML exist, including R versus S optical isomers and GML with lauric acid ester linked in the 1/3 or 2 position of glycerol. These compounds were tested in the following way. The R form of GML was available, but the S form was not. Thus we compared the purified R form to the mixture of R and S. Similarly, we purchased the purified 2 position lauric acid form of GML, and thus we were able to compare this form to the GML R and S mixture. These GML forms were evaluated for activity against both group A and group B streptococci as two highly sensitive organisms to GML antibacterial activity. For *Streptococcus pyogenes* (strain 594) (Figure 10) and *Streptococcus agalactiae* (strain MNSI) (data not shown), both the R form and mixture of R and S forms had the same antibacterial activity. The GML form with lauric acid in the 2 position was 2-fold more active than the mixture of GML forms (Figure 10). The data suggest that GML activity does not depend on chirality, but in a minor way depends on the position of the lauric acid.

### Spectrum of GML Antibacterial Activity

We examined a large collection of bacterial isolates for growth inhibition by GML (Table 1). Both gram-positive and gram-negative bacteria were inhibited from growing under optimal growth conditions for each organism tested. Only two major groups of potentially pathogenic organisms were not inhibited by GML, the large group of *Enterobacteriaceae* and *Pseudomonas aeruginosa*, even at concentrations of 5000 μg/ml. Interestingly, *Burkholderia cenocepacia*, which used to be named *Pseudomonas cepacia* and is related to *Pseudomonas aeruginosa*, was killed by GML at concentrations of 500 μg/ml. Mycobacterial species typically produce large amounts of complex fatty acids [16]. However, these organisms were killed by GML at concentrations of ≥50 μg/ml. In addition to inhibiting the growth gram-positive bacteria, GML inhibited exotoxin production independently from inhibition of growth for all such organisms tested (*Staphylococcus aureus, Streptococcus pyogenes, Streptococcus agalactiae*, groups C, F, and G streptococci, and *Clostridium perfringens*). The most susceptible organisms to killing by GML were *Peptostreptococcus species, Clostridium perfringens, Bordetella bronchiseptica*, and *Campylobacter jejuni*, all being killed by GML (1 μg/ml).

**Table 1:** Spectrum of antibacterial activity of GML.

Bacterium	Gram or Other Stain	Oxygen Tolerance	Strains Tested	Average Bactericidal Concentration (μg/ml)
*Staphylococcus aureus*	Positive	Aerobe	54	300
*Streptococcus pyogenes*	Positive	Aerotolerant Anaerobe	4	30
*Streptococcus agalactiae*	Positive	Aerotolerant Anaerobe	3	30
Group C Streptococcus	Positive	Aerotolerant Anaerobe	1	30
Group F Streptococcus	Positive	Aerotolerant Anaerobe	1	20
Group G Streptococcus	Positive	Aerotolerant Anaerobe	1	50
*Streptococcus suis*	Positive	Aerotolerant Anaerobe	1	50
*Streptococcus sanguinis*	Positive	Aerotolerant Anaerobe	1	50
*Streptococcus pneumoniae* Serotype III	Positive	Aerotolerant Anaerobe	2	10
*Enterococcus faecalis*	Positive	Aerotolerant Anaerobe	1	100
*Listeria monocytogenes*	Positive	Aerobe	1	50
*Bacillus anthracis*Sterne	Positive	Aerobe	1	50
*Bacillus cereus*	Positive	Aerobe	1	50
*Peptostreptococcus species*	Positive	Anaerobe	1	1
*Clostridium perfringens*	Positive	Anaerobe	1	1
*Neisseria gonorrhoeae*	Negative	Aerobe	1	20
*Haemophilus influenzae* Non-typable	Negative	Aerobe	2	50
*Gardnerella vaginalis*	Negative	Aerobe	2	10
*Campylobacter jejuni*	Negative	Aerobe	1	1
*Bordetella bronchiseptica*	Negative	Aerobe	1	1
*Pseudomonas aeruginosa*	Negative	Aerobe	1	Not Susceptible
*Burkholderia cenocepacia*	Negative	Aerobe	1	500
*Pasteurella multocida*	Negative	Aerobe	1	500
*Prevotella melaninogenica*	Negative	Anaerobe	1	50
*Bacteroides fragilis*	Negative	Anaerobe	2	50
*Fusobacterium species*	Negative	Anaerobe	1	50
*Escherichia coli*	Negative	Aerobe	2	Not Susceptible
*Salmonella minnesota*	Negative	Aerobe	1	Not Susceptible
*Enterobacter aerogenes*	Negative	Aerobe	1	Not Susceptible
*Proteus vulgaris*	Negative	Aerobe	1	Not Susceptible
*Shigella sonnei*	Negative	Aerobe	1	Not Susceptible
*Klebsiella pneumoniae*	Negative	Aerobe	1	Not Susceptible
*Mycobacterium phlei*	Acid Fast	Aerobe	1	100
*Mycobacterium tuberculosis*	Acid Fast	Aerobe	1	100
*Mycoplasma hominis*	Cell Wall deficient	Aerobe	1	1

### Use of accelerants to increase GML activity against Enterobacteriaceae and Pseudomonas aeruginosa

**Figure 11:**
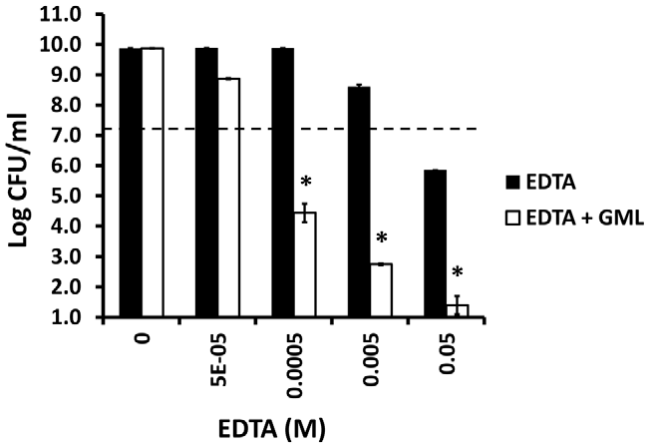
Ability of EDTA to synergize with GML in antibacterial activity against *Escherichia coli.* * Escherichia coli* Watson strain was incubated with EDTA or EDTA + GML (100 μg/ml) for 24 hours and then plate counts used to determine CFUs/ml. GML (5000 μg/ml) alone did not inhibit *Escherichia coli* growth. * indicates mean for EDTA + GML differs significantly from mean for EDTA alone at p<0.001. Dashed line indicates starting inoculums size.

Our prior studies suggested that the intact LPS layer of *Enterobacteriaceae* protected this family of organisms from GML [2]. Thus, we attempted to partially disrupt the LPS layer in these organisms and evaluate GML activity. In the first set of studies we tested the effect of EDTA on GML (100 μg/ml) activity against *Escherichia coli* (Figure 11). GML as expected exerted no measurable antibacterial activity against *Escherichia coli* at concentrations up to 5000 μg/ml (data not shown). EDTA alone exhibited bacteriostatic and bactericidal activity against *Escherichia coli*, dependent on EDTA concentration. The combination of GML with EDTA showed increased activity when used in combination with GML at a concentration of 100 μg/ml.

**Figure 12:**
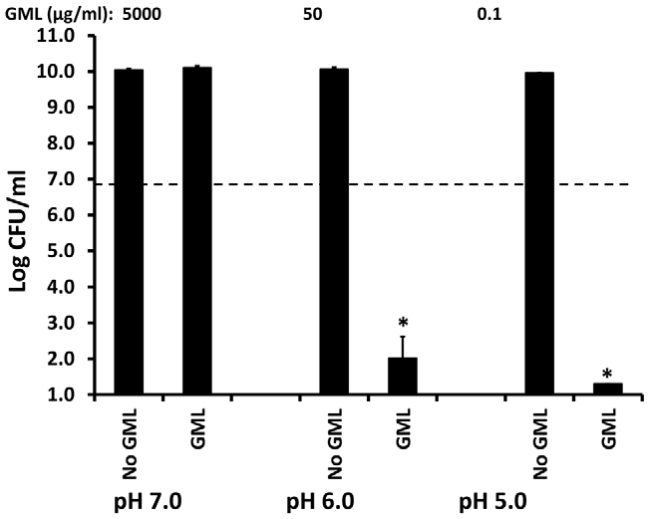
Effect of pH on GML activity against *Escherichia coli* Watson strain. Todd Hewitt broth was buffered to pH 7.0, 6.0, and 5.0. Susceptibility of *Escherichia coli* to GML at the indicated pH was measured in 24 hour assays. CFUs/ml were determined by plate counts. * indicates means of No GML samples and GML samples differ significantly at p<0.001. Dashed line indicates the inoculum size.

Because EDTA made *Escherichia coli* more susceptible to GML than in its absence, we hypothesized that protonating the *Escherichia coli* surface may increase GML activity through repelling divalent cations. *Escherichia coli* was highly susceptible to GML at pH of 6, with GML (50 μg/ml) being bactericidal, despite not being susceptible at pH 7.0 to even 100 times the GML concentration (Figure 12). *Escherichia coli* was even more susceptible to GML at pH of 5, with GML (0.1 μg/ml) being bactericidal. With each unit drop in pH, it appeared that *Escherichia coli* became 500 times more susceptible to GML. We also tested GML susceptible gram-negative bacteria, such as *Haemophilus influenzae* nontypable organisms, and these organisms were similarly 500-fold more susceptible to GML with each unit drop in pH (data not shown).

**Figure 13:**
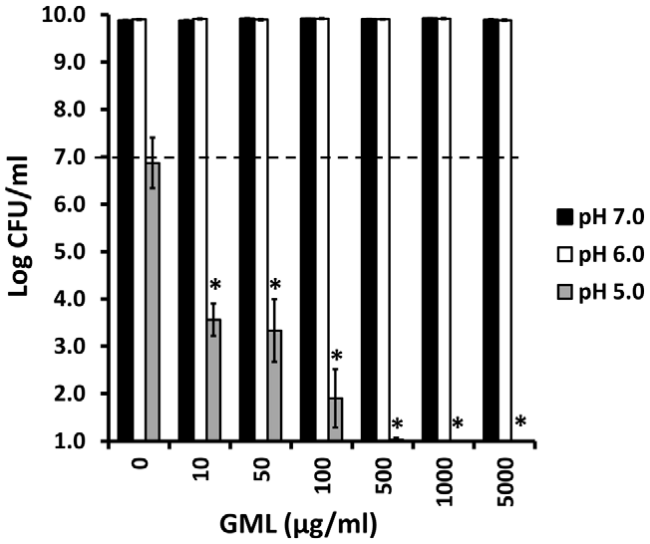
Effect of pH on GML activity against *Pseudomonas aeruginosa.* Todd Hewitt broth was buffered to pH 7.0, 6.0, and 5.0. Susceptibility of *Pseudomonas aeruginosa* PA01 to GML at the indicated pH was measured in 24 hour assays. CFUs/ml were determined by plate counts. * indicates sample means differ significantly from the no GML control at p<0.001. Dashed line indicates the inoculum size.

We also tested *Pseudomonas aeruginosa* for the effect of pH on GML activity against this organism (Figure 13). *Pseudomonas aeruginosa* was not susceptible to GML at either pH 6.0 or 7.0. However, the organism did not grow well at pH 5.0, but GML (50 μg/ml) at pH 5.0 was highly bactericidal for *Pseudomonas aeruginosa.*

**Figure 14:**
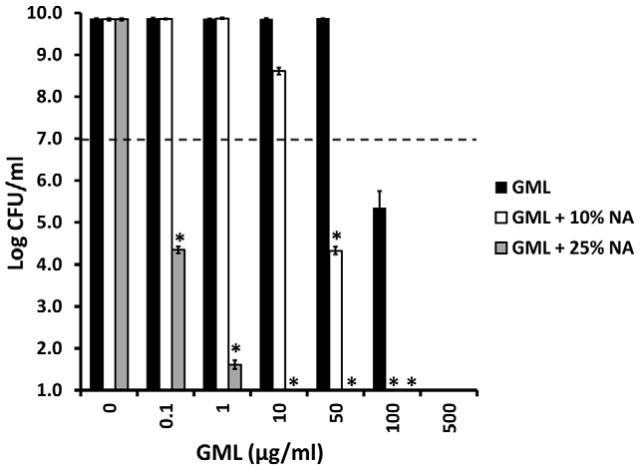
Effect of a nonaqueous (NA) gel on GML antibacterial activity against *Staphylococcus aureus* MN8. Non-aqueous gel was diluted to 25% and 10% with Todd Hewitt broth. GML and *Staphylococcus aureus* MN8 were added, and cultures were incubated for 24 hours. CFU/ml were determined by plate counts. Dashed line indicates the inoculum size. * indicates mean differs significantly from the GML alone control at p<0.001.

It is unlikely that GML will be used as an antibacterial agent as suspended in aqueous solutions do to its solubility limit of 100 μg/ml in aqueous solutions at 37°C. However, our experience has been that GML exhibits antibacterial activities even at concentrations that exceed 100 μg/ml, despite the lack of solubility in aqueous solutions at that concentration [2]. For ease of use, we developed a non-aqueous, biocompatible delivery system that could be used to solubilize GML up to concentrations of 50,000 μg/ml. This delivery system is related to K-Y Warming® gel, containing 73.55% (w/w) propylene glycol, 25% (w/w) polyethylene glycol 400 NF, and 1.25% (w/w) hydroxypropyl cellulose. This solution has been shown to be safe for humans and non-human primates in chronic use studies for up to six months [17]. The gel had no deleterious effect on normal flora lactobacilli, but did effectively reduce both *Candida* and *Gardnerella vaginalis* [11]. We tested this delivery vehicle with GML for ability to kill *Staphylococcus aureus* MN8 (Figure 14). Both delivery vehicle alone and GML in delivery vehicle were bactericidal, preventing determination of potential synergy between vehicle and GML. We therefore tested GML activity when vehicle alone and GML in vehicle were diluted with various volumes of Todd Hewitt broth. GML in the 25% and 10% non-aqueous delivery vehicle had significantly greater antibacterial activity than vehicle alone (no activity) or GML alone diluted comparably in Todd Hewitt broth. GML in 25% non-aqueous delivery vehicle had approximately 5000-fold greater activity than GML alone, and GML in 10% aqueous delivery vehicle had approximately 10-fold greater activity than GML alone.

## Discussion

We performed extensive studies of the potential ability of GML to be used as a topical bactericidal agent and biocide for decontamination of environmental sources of potential pathogens. These studies show that GML has potent bactericidal activity against a myriad of organisms that cause human illnesses. Previous studies demonstrated that GML has strong activity against gram-positive bacteria, notably gram-positive cocci [2], [4]. Our studies confirm and extend those previous findings to include additional organisms, including anaerobes. Additionally, prior studies demonstrated that GML inhibited exotoxin production by gram-positive bacteria at GML concentrations that were not growth-inhibitory [2], [4]. We also confirmed those findings but extended the studies to include inhibition of exotoxin production by pathogenic clostridia.

There is general agreement in the literature that the major effect of GML to inhibit gram-positive exotoxin production is prevention of signal transduction through two-component systems to interfere with transcription [2], [3], [4], [9], [18]. This has been shown for TSST-1 and α-toxin in vitro and in vivo and has led to incorporation of GML into certain tampons to reduce the risk of TSS. Exotoxin production by *Bacillus anthracis* is also inhibited by GML, independent of inhibition of bacterial growth, and this inhibition has been suggested also to depend on interference with two-component system signal transduction [3]. In studies by Ruzin and Novick it has been suggested that the ability of GML to inhibit exotoxin production by GML may in fact be due to lauric acid, as both compounds have this effect, and GML can be cleaved by GEH to lauric acid [9]. We have confirmed that both GML and lauric acid have this activity. However, our studies show that GML inhibits exotoxin production by organisms that do not make GEH, indicating GML does not require cleavage to lauric acid for activity. In addition, GML inhibits exotoxin production at doses that are 200-fold lower than lauric acid. Thus, the major antibacterial effect of GML is much greater than lauric acid, but both molecules appear to alter signal transduction to inhibit exotoxin production. The exact two-component systems that are most sensitive to GML's ability to inhibit exotoxin production remain unclear. At this time, it has been shown that GML affects the *agr* regulatory system, but this is only one of many two component system that regulates exotoxin production [4].

It also remains unclear how GML kills bacteria in less than 15 min, but the effect almost certainly depends on interaction with the bacterial plasma membrane since we detect GML associated with bacterial plasma membranes but not cytoplasm (unpublished data). There are multiple possibilities to explain the killing effect that include interference with two-component systems. *S. aureus* has 16 identified two-component systems, and one of these, designated WalK/R, is essential for microbial viability [19]. As noted above, at least *agr* is inhibited by GML, and this effect likely contributes to inhibition of exotoxin production. If two-component systems differentially are inhibited by GML, it is possible that inhibition of exotoxin occurs at low GML doses after systems such as *agr* are affected, and then at high GML doses, WalK/R is inhibited, leading to death of the organisms.

Although direct inhibition of two-component systems is a possible mechanism of GML killing of bacteria, there are other important possibilities, including indirect inhibition of the same two-component systems. Among these possibilities are dissipation of the bacterial plasma membrane potential and pH gradients across the membranes. A novel class of agents that have these effects include tetramic acids, for example those produced by *Pseudomonas aeruginosa* and certain lactobacillus strains [20], [21], [22]. Tetramic acids made by these organisms contain a 2,4 pyrrolidinedione ring and a 12 carbon side chain. Their properties include broad spectrum antibacterial effects and anti-inflammatory activities, in many ways paralleling those of GML. It is possible the similarities between tetramic acids and GML may explain why *Pseudomonas aeruginosa* and lactobacilli are highly resistant to GML antimicrobial activities. For *Pseudomonas aeruginosa*, tetramic acids are important for the homoserine lactone quorum sensing system [20], [21]. Interestingly, the organism as grown in the presence of high concentrations of GML (>2000 μg/ml) at pH 7.0 appears to have up-regulated production of numerous virulence factors including pigments, consistent with effects associated with activation of the quorum sensing system.

We have proposed that the major use of GML will include its function as a topical microbicide [6], [11], [23]. We have previously shown that GML reduces vaginal *Candida* and *Gardnerella vaginalis* in women, while at the same time not affecting normal flora lactobacilli [11]. In other studies we showed that GML reduces vaginal *Staphylococcus aureus* and *Streptococcus agalactiae* [23]. All of these organisms vaginally are likely to be growing as biofilms on tampons or on vaginal mucosal surfaces.

Biofilms present a challenge in infections due to antibiotic resistances within biofilm communities and inaccessibility of biofilms microbes to the immune system [1]. In our studies, we have demonstrated that GML interferes with biofilm formation, using as test organisms one gram-positive and one gram-negative organism. We showed GML prevents biofilm formation on tampons, cellulose acetate, which is the best surface we have found for biofilm formation, and plastic 96-well microtiter plates. Additionally, GML has the ability to loosen established biofilms from surfaces, such as on the surface of microtiter plates. Importantly, the bacteria within the detached biofilms are killed.

In the course of our studies of TSST-1 production within tampon sacs [12], models for what may happen in vivo in women with use of tampons, we demonstrated that as much as 18 mg/ml of TSST-1 may be present in the tampon sacs. With an average fluid uptake in 16 hours of 5.5 ml, this translates into nearly enough TSST-1 to cause illness in 10^6^ individuals. Thus, TSST-1 is produced in extremely high concentrations by the USA200 lineage that is most associated with mucosal surface-derived TSS [24]. These data are important because they are in contrast to a recent study by DeLeo et al. that suggested that a mutation in *agr* reduces the virulence of USA200 strains [13]. This is clearly not the case with TSST-1 production by these strains, and it is well-established that TSST-1 is the principal cause of mucosal TSS, including menstrual, tampons associated illness [24], [25]. It has also been established that TSST-1 production is tightly regulated in vitro by *agr* [26]. Thus, it appears that either *agr* no longer regulates TSST-1 production in USA200 strains, or the mutation observed by DeLeo et al. [13] is in a region that does not strongly affect virulence factor production. Incidentally, in vivo in used tampons from women, we have observed up to 100 μg of TSST-1 that was produced generally in only a few hours [27].

Many antibiotics have specific bacterial targets. In having their activities, this often implies that chirality is important in activity with either R or S form having major activity, but not both. In our studies, we observed that both the R and mixture of R and S forms have comparable activity that suggests that both R and S are active. If membrane-bound histidine kinases of two-component systems are affected by GML, the effect may be through alteration of conformation by altering the plasma membrane, rather than interaction with the histidine kinase itself. There must be something special about GML's interaction with the plasma membrane since it is well-recognized that GML has greater antibacterial activity than glycerol monoesters that have shorter or longer fatty acid chains [28], [29]. We do not know why GML with lauric acid in the 2 position has slightly greater antibacterial activity than GML with lauric acid in the 1 or 3 position.

We have performed extensive studies, without success, in attempt to produce resistance in *Staphylococcus aureus* to GML. For most antibiotics, this organism develops resistance either through target modification or acquisition of a gene encoding a protein that allows the organism to bypass or inactive the antibiotic. Despite one year of passage on sub-growth-inhibitory concentrations of GML, we did not identify even a single mutant that developed resistance to GML. Likewise we did not isolate mutants that had up-regulated GEH production that inactivates GML. In prior studies in which we tested susceptibility of *S. aureus* to GML by a disk diffusion method, which would allow for exposures to a gradient of GML concentrations that provides selective pressure, no GML resistant mutants were seen [17]. Collectively, these data suggest that there are likely to be multiple bacterial targets for GML, such that resistance is unlikely to occur.

Our studies also show that GML has a broad range of activity against bacteria. With the exception of *Enterobacteriaceae* and *Pseudomonas aeruginosa*, all potential bacteria tested were susceptible to GML. This includes both aerobes and anaerobes, and gram-positive, gram-negative, and non-gram-staining bacteria. The fact that *Mycoplasma* are killed by GML indicates the cell wall is not a target of GML, since these organisms lack cell walls. This is consistent with a prior in vivo study that shows that GML also can be used to kill *Trichomonas* and *Candida*, neither of which has a peptidoglycan cell wall [11]. In general, gram-positive and gram-negative bacteria without intact LPS were equivalently susceptible to GML, with those lacking GEH or a related esterase being 10-fold more susceptible. It is interesting that GML kills *Pasteurella multocida* and *Burkholderia cenocepacia* but does not kill related *Enterobacteriaceae* and *Pseudomonas*, respectively. These differences suggest that LPS organization is different among the organisms, as we have shown that organisms with LOS are uniformly killed by GML, whereas those with intact LPS are resistant [2]. Of special interest is that mycobacteria are killed by GML. These organisms are characteristically difficult to kill due to the presence of complex waxes in their cell walls.

We have suggested that GML may have use as a topical microbicide, showing that the compound reduces SIV transmission in non-human primates [6]. GML has also been extensively studied in chronic (6 month) in vivo vaginal safety studies in non-human primates. No toxicity or vaginal inflammation in any animal was observed over the 6 month study period as evaluated by repeated colposcopy and biopsy studies [17]. We have shown that GML stabilizes mammalian cells against toxicity due to multiple bacterial exotoxins and hypotonic solutions [30]. GML also kills *Gardnerella vaginalis* in vitro and in vivo vaginally, an organism that predisposes women to HIV transmission [11]. Additionally, we have shown that GML stabilizes mucosal surfaces without killing epithelial cells, preventing inflammation that also predisposes to HIV transmission [6], [23], [30]. This has been shown both in vitro and vaginally in both non-human primates and women using tampons. There are many other potential uses for GML, but for these uses to be maximized, it may be necessary to identify agents that increase GML activity against *Enterobacteriaceae* and *Pseudomonas aeruginosa.*

As noted above both of these groups of organisms cause disease in humans, and both are highly resistant to GML. The resistance of *Enterobacteriaceae* to GML is clearly dependent on having an intact LPS [2]. This suggested to us that agents that disrupt the integrity of LPS would increase GML activity. Three agents were studied: 1) reduced pH that would be expected to protonate the bacterial surface and repel calcium and magnesium ions that help maintain LPS integrity, 2) use of calcium and magnesium chelators, and 3) agents disrupt the integrity of interaction of LPS with phospholipids in the outer membrane, such as non-aqueous GML delivery vehicles. All three of these agents, which are categorized as accelerants, increase GML activity. It is interesting that for each one pH unit reduction, there was a 500-fold drop in GML activity against *Enterobacteriaceae* such as *Escherichia coli.* This means that at pH 4.0 compared to pH 7.0 GML was over 10^8^ more active against the organism.

It remains unclear why *Pseudomonas aeruginosa* is not susceptible to GML, but as noted above part of the resistance may be due to similarities in tetramic acids involved in the organisms' quorum sensing systems and GML. The organism does not have the *Enterobacteriaceae* LPS, and thus we hypothesized that GML would easily kill the organism. This is clearly not the case. Indeed, in performing the experiments, it appeared that at pH 7.0 GML caused highly significant up-regulation of virulence factor production (data not shown). Pigment production was clearly up-regulated, as cultures turned visibly green with growth in GML. Like *Enterobacteriaceae, Pseudomonas aeruginosa* was killed by GML in acidic pH, although the organism itself exhibited greater susceptibility to acid pH alone.

In conclusion, this study has examined in detail the antibacterial activities of the generally recognized as safe compound GML. GML has potential use as a broadly-acting topical microbicide on human and animal mucosa and skin surfaces, and in environmental sources of human and animal infections. We refer to GML as a dual acting anti-infective because it kills bacteria, while at the same time stabilizing mucosal and skin surfaces to prevent inflammation that may be required for microbial infection to occur. GML has many advantages to other agents because of its safety record and because resistance is unlikely to develop.

## Materials and Methods

### Bacteria

*Staphylococcus aureus* MN8 is a typical menstrual TSS organism [5]. The organism is classified as a pulsed-filed gel electrophoresis clonal type CDC USA200 organism; it is methicillin-sensitive and produces TSST-1. *Staphylococcus aureus* 128 is a CA-MRSA USA200 strain that produces TSST-1. *Streptococcus pyogenes* strain T25_3_curedT12 was originally obtained from a patient with scarlet fever [31]. This strain produces SPE A. *Streptococcus pyogenes* strain 594 is highly sensitive to GML; the strain produces SPE A [32]. *Streptococcus agalactiae* strain MNSI was isolated from a patient with neonatal sepsis. *Escherichia coli* strain Watson was isolated from a female patient with a urinary tract infection. *Pseudomonas aeruginosa* PA01 was kindly provided by Dr. C. Mohr, University of Minnesota, Minneapolis, MN. Non-typable *Haemophilus influenzae* was kindly provided by the late Dr. Scott Giebink, University of Minnesota, Minneapolis. All strains listed in Table 1 were either clinical isolates or were strains listed above. All strains were cultured in media that supports their optimal growth. Anaerobes were cultured stationary in an anaerobic chamber or GasPak jar (Becton Dickinson, Sparks, MD). Aerobes were cultured in a standard incubator with aeration (shaking at 200 revolutions/min), and aerotolerant anaerobes (streptococci) were cultured stationary in the presence of 7% CO_2_. *Neisseria gonorrhoeae* was not culturable on standard laboratory media. Thus, for determination of GML inhibition of growth of this organism, *Neisseria* were cultured on chocolate agar plates, then washed off the plates with PBS, and finally suspended in Todd Hewitt media for designated time periods with gentle shaking (100 revolutions/min) in the presence of 7% CO_2_. The organism was plated onto chocolate agar plates for determination of CFUs/ml.

### GML and derivatives and lauric acid

GML was purchased from Colonial Chemical Inc, South Pittsburg, Tennessee. The R form of GML was purchased from Fontarome Chemical Company, Milwaukee, WI. The 2 form of GML was purchased from Indofine Chemical Company, Hillsborough, NJ. Lauric acid was purchased from Sigma-Aldrich, St. Louis, MO.

### Assays for bacterial growth and superantigen production

For experimentation, other than in Table 1, bacteria were cultured in Todd Hewitt broth (Difco Laboratories, Detroit MI). *Staphylococcus* aureus, *Haemophilus influenzae*, *Escherichia coli*, and *Pseudomonas aeruginosa* strains were cultured at 37°C with 200 revolutions/min shaking for designated periods of time. *Streptococcus pyogenes* and *Streptococcus agalactiae* strains were cultured stationary at 37°C in the presence of 7% CO_2_. Initial inocula were estimated by determining absorbance at 600 nm wavelength, and then verification by plate counts. For *Staphylococcus aureus* strains, an absorbance of 1.0 routinely correlated with plate counts of 1×10^9^/ml. For streptococci, an absorbance of 1.0 correlated with 5×10^8^ CFUs/ml. For both *Escherichia coli* and *Pseudomonas aeruginosa*, an absorbance of 1.0 correlated with 1×10^8^ CFU/ml. Plate counts on Todd Hewitt or blood agar plates were used for determination of CFUs/ml. Quantitative Western immunoblots were used for determination of superantigens [33].

### Biofilm studies

Tampon sacs were set up as described previously [12]. Briefly, cellulose acetate dialysis tubing (12,000–14,000 molecular weight cut-off) (Spectra-Por, Rancho Dominguez, CA) was tied off on one end and inoculated with approximately 10^7^ *Staphylococcus aureus* in 0.1 ml volumes. Regular absorbency tampons were placed within the dialysis tubing, and the tampon sacs were submerged beneath Todd Hewitt broth media containing 0.8% agar. The open end of the tampon sacs remained above the liquid to prevent direct inoculation of the internal contents with culture media. When the agar had solidified, the tampon sac cultures were incubated at 37°C stationary for designated periods of time.

For determination of CFUs/ml and TSST-1 production, the tampon sacs were removed from the agar, they were sliced open and weighed to determine fluid gain (it was assumed 1 ml weight gain corresponded to 1 gm weight gain), and bacteria and TSST-1 were eluted by addition of the tampons to syringes with 10 ml of PBS and expressing the fluids. CFUs/ml were determined by plate counts. TSST-1 was quantified by first concentrating the eluted fluids by addition of 4 volumes of absolute ethanol, followed by resolubilization in distilled water, and Western immunoblot analysis [33].

In other biofilm studies, bacteria were added to 96-well microter plates (Falcon Plastics, Oxnard, CA). The plates were incubated for up to 48 hours with *Staphylococcus aureus* or *Haemophilus influenzae*. The plates were incubated stationary at 37°C. Subsequently, CFUs/ml were determined by plate counts after briskly pipetting well contents up-and-down three times. Then, wells were washed three times with PBS with brisk pipetting at each addition of PBS. Finally, wells were treated with crystal violet [15], subsequently washed three times with PBS, and absorbance at 595 nm wavelength determined after solubilization of residual crystal violet with absolute ethanol.

### Tests of accelerants

EDTA was purchased from Sigma Aldrich and added to Todd Hewitt media at indicated concentrations. The non-aqueous gel used in accelerant studies was made as follows: Propylene glycol (Gallipot, St. Paul, MN) (73.55% w/w) was mixed with polyethylene glycol (Gallipot) (25% w/w) and hydroxypropyl cellulose (Gallipot) as a gelling agent (1.25% w/w). The compounds were heated to 65°C for solubilization of components and for solubilization of GML.

### Tests for development of staphylococcal resistance to GML

*S. aureus* MN8 was used as the test organism in year-long studies. We first established that the MBC of GML in Todd Hewitt agar plates for this organism was approximately 100 μg/ml. This was accomplished by plating approximately 10^7^ CFUs of MN8 in 0.1 ml volumes in triplicate on Todd Hewitt plates containing 0, 12.5, 25, 50, 100, and 200 μg/ml GML. The organism grew to confluence on the 0, 12.5, 25, and 50 μg/ml plates in 24–48 hours, but no growth of the organism was observed on the Todd Hewitt plates containing 100 and 200 μg/ml GML over the 48 hour test period. Also, through use of serial 10-fold dilutions of *S. aureus* MN8, that there was no significant CFU differences among the GML concentrations between 0 and 50 μg/ml. We thus chose GML (50 μg/ml; 0.5 x MBC) as a sub-inhibitory concentration to provide selective pressure for development of mutants over a one-year time period that could grow on the GML (100 μg/ml; 1 x MBC) Todd Hewitt agar plates in 48 hours. The organism was diluted and cultured in triplicate on GML (50 μg/ml) Todd Hewitt plates such that 50–200 colonies would grow in 48 hours at 37°C. After 48 hours of growth, the plates were placed at 4°C for the remainder of the week. Then, the colonies were washed off the plates and CFUs adjusted to approximately 1×10^10^/ml. These organisms were then plated onto fresh GML (50 μg/ml and 100 μg/ml) Todd Hewitt plates by spreading 0.1 ml (approximately 1×10^9^ CFUs) in triplicate per plate. Growth was monitored over the next 48 hours. The process was repeated weekly for 52 weeks. Controls consisted of performing the same assays with growth on Todd Hewitt agar plates without GML.

We also developed an assay that would allow us to evaluate enhanced production of GEH as a result of growth on GML (50 μg/ml) plates. In this assay the GML (50 μg/ml) plates that were placed at 4°C were examined directly for GEH production. GML (50 μg/ml) forms white crystals on Todd Hewitt agar at 4°C, whereas the GML cleavage products do not. Thus, enhanced GEH production, if selected for over the one year period, could be visualized by direct measurement of increased clear zones around colonies in a white crystalline background.

### Statistics

Means and standard deviations were determined for all experiments. In studies to compare means between treatment groups, Student's *t* test analysis was used to determine significant differences.
